# Global epidemiology of *Helicobacter pylori iceA* genotypes: heterogeneity and virulence implications

**DOI:** 10.1128/spectrum.02024-25

**Published:** 2026-05-29

**Authors:** Yayi Ren, Jia Xie, Long Zhou, Zelin Zhang, Tingting Xia, Jie Ning, Chao Wu

**Affiliations:** 1 Department of Laboratory Medicine, The Eighth Affiliated Hospital of Sun Yat-Sen University 575842 , Shenzhen, China; 2 School of Biology and Biological Engineering, South China University of Technology 26467, Guangzhou, China; Taichung Veterans General Hospital, Taichung, Taiwan

**Keywords:** *Helicobacter pylori*, virulence factors, *iceA* genotype, geographical heterogeneity

## Abstract

The contact-induced epithelium gene A (*iceA*), with allelic variants *iceA1* and *iceA2*, remains a controversial virulence factor in *Helicobacter pylori* due to inconsistent clinical outcomes. In this study, we analyzed 1,427 *H. pylori* genomes from the NCBI database and 200 clinical isolates from Shenzhen, China, to elucidate the global distribution of *iceA* genotypes and their associations with other virulence factors, bacterial gene expression, and the relationship with gastric diseases. We found that *iceA* genotypes varied significantly across regions, with *iceA1* predominating in Asia and Europe, and *iceA2* being more prevalent in North America. This suggests adaptive evolution to regional host environments. The association between *iceA* genotypes and *cagA* status also exhibited regional patterns. Additionally, *iceA1* was linked to higher *ureA* expression, potentially enhancing acid resistance and colonization potential (*P* < 0.05). Importantly, patients infected with *iceA1* genotype strains were more likely to present with gastric erosion (*P* < 0.05); however, they showed no association with peptic ulcer disease in any region. These findings highlight the geographical heterogeneity of *iceA1* genotypes and their potential role in *H. pylori*-related gastric diseases.

**IMPORTANCE:**

Our study found that the iceA1 genotype of *Helicobacter pylori*, which is relatively common in Asia, is associated with increased urea hydrolysis capacity and shows a specific association with erosive gastritis in Chinese patients. These findings suggest that iceA1 may potentially serve as an early biomarker for the identification of patients at risk of progressive gastric damage. Clinically, detecting iceA1 could improve risk stratification in high-prevalence regions, guiding more targeted monitoring and early intervention strategies. This research supports incorporating bacterial genotyping into clinical practice to optimize personalized treatment and prevent disease progression in susceptible populations.

## INTRODUCTION

*Helicobacter pylori* is a spiral-shaped bacterium first identified by Barry Marshall and Robin Warren in 1982 (1, 2). This bacterium neutralizes gastric acid through urea hydrolysis, thereby facilitating its colonization in the acidic gastric milieu and inducing chronic inflammation. While nearly all individuals infected with *H. pylori* develop chronic gastritis, the majority remain asymptomatic (3, 4). However, a subset of infected individuals may experience more severe clinical outcomes, including peptic ulcer disease in approximately 10%, gastric cancer in 1%–3%, and mucosa-associated lymphoid tissue lymphoma in 0.1% (5, 6). The clinical outcomes of *H. pylori* infection are largely determined by the presence and type of virulence factors, which modulate disease severity and progression. The *H. pylori* genome contains multiple virulence genes with diverse functions, and different strains carry distinct virulence genes that are essential for the development and progression of associated diseases.

Elucidating the full spectrum of *H. pylori* virulence genes and their interactions is critical for enhancing the diagnosis, treatment, and prognosis of related diseases. The urease protein enables *H. pylori* to neutralize gastric acid and colonize the gastric mucosa (7). Motility genes, including flagellin A (*flaA*) and flagellin B (*flaB*), encode proteins that allow the bacterium to penetrate the gastric mucus layer and reach the epithelial surface (8). Critical virulence factors, such as the vacuolating cytotoxin (encoded by *vacA*) and the cytotoxin-associated gene protein (encoded by *cagA*), contribute to pathogenesis; VacA protein induces vacuolation and disrupts cell membranes (9), while CagA interferes with host cell signaling, leading to morphological changes and abnormal proliferation (10). Collectively, these genes enable *H. pylori* to colonize, persist, and cause disease, with clinical manifestations ranging from gastritis to peptic ulcers and gastric cancer (11–13). However, the roles of some virulence genes remain less clear, and their association with pathogenicity is controversial. Particularly, the contact-induced epithelium gene A (*iceA*) has been implicated in disease, but its exact function and contribution to virulence remain unclear and require further investigation (14, 15).

The *iceA* gene was identified in *H. pylori* following exposure to gastric epithelial cells (14). Two major variants, *iceA1* and *iceA2*, have been characterized, exhibiting significant genetic sequence differences. Although these two genotypes occupy the same genomic locus, they diverge markedly in nucleotide sequence and length, with *iceA1* being notably longer (about 700 bp) than *iceA2* (about 300 bp). The *iceA* gene is located adjacent to *hpyIM*, which encodes a DNA methyltransferase homologous to the cognate methylase in *Neisseria lactamica*, and the *iceA1* gene product shows approximately 52%–57% similarity to the NlaIII protein from *Neisseria lactamica* (16, 17). This gene arrangement suggests that *iceA1* and *hpyIM* form a restriction-modification system akin to the NlaIII system in *N. lactamica*, supporting functional homology. However, most *H. pylori iceA1* genes harbor various mutations, including frameshift deletions and insertions. These mutations prevent the *iceA1* gene from being translated into a functional restriction endonuclease protein with nucleic acid cleavage activity (17, 18). Despite the high prevalence of *iceA1* in *H. pylori* strains, the exact role of these mutated *iceA1* genes remains unclear. Some hypotheses suggest that even non-functional *iceA1* gene products or truncated proteins might play roles in bacterial adaptation or in modulating host immune responses, but this requires further investigation (19). In contrast, *iceA2* has not been reported to share homology with any known genes.

According to research reports, strains carrying the *iceA1* genotype induce inflammatory responses in mucosal epithelial cells, increasing IL-8 secretion and neutrophil infiltration (20). These effects exacerbate tissue damage and are associated with more severe clinical outcomes, such as peptic ulcers; in contrast, the *iceA2* allele shows no significant correlation with disease manifestations or pathogenic mechanisms (14, 21). The *iceA* genotypes also display distinct geographic and population-specific distributions, likely reflecting adaptations to diverse host environments or pathogenic strategies (16, 22–24). However, most previous studies were limited to single countries or regions, resulting in a narrow scope of investigation. Besides, studies from some regions report discrepancies regarding the role of *iceA* in *H. pylori* pathogenicity; for example, research in Japan and Mexico has shown no significant association between *iceA1* and *H. pylori*-induced gastric ulceration (25, 26). These conflicting findings suggest that the function of *iceA* may not be universal but is likely modulated by additional factors. Such divergences underscore the gene’s complex operational mechanisms and, more importantly, reveal critical deficiencies in our current conceptual framework, which may oversimplify its role. Therefore, a comprehensive, global-scale analysis that controls for population-specific genetic backgrounds and environmental influences is urgently needed to reconcile these contradictions and build a unified model.

Furthermore, the molecular mechanisms of *iceA*’s function and its potential regulatory interactions with other *H. pylori* virulence genes remain largely unexplored. These knowledge gaps highlight the necessity for large-scale, multi-population studies to clarify *iceA*’s epidemiological patterns, resolve contradictory clinical associations, and elucidate its potential role in *H. pylori*’s adaptive strategies.

To address these knowledge gaps and geographical inconsistencies, we designed a multicenter study to: characterize the global epidemiology of *iceA* variants and their disease-specific associations across different populations and investigate the correlations between *iceA* genotypes and major virulence factors. This global perspective is essential for identifying region-specific patterns and universal trends, providing a robust data set for statistical analysis, and accounting for geographical variations in *H. pylori* strains and host factors. Our study assessed the distribution of *iceA* and investigated its involvement in *H. pylori* virulence, including its synergistic interactions with other pathogenic factors and correlation with bacterial gene expression. This comprehensive investigation will enhance our understanding of *H. pylori* pathogenicity and aims to uncover new targets and strategies for the diagnosis and treatment of *H. pylori*-associated diseases, thereby providing a more complete picture of *iceA*’s significance in infection outcomes and function.

## MATERIALS AND METHODS

### Study population and design

This population-based cross-sectional study was conducted to characterize the global prevalence of *H. pylori iceA* subtypes and their virulence in different regions. A total of 200 clinical *H. pylori* strains were isolated from patients undergoing upper gastrointestinal endoscopy at the Eighth Affiliated Hospital of Sun Yat-sen University in Shenzhen between December 2022 and June 2024. Participants meeting all of the following criteria were enrolled: (i) age between 18 and 70 years; (ii) no use of proton pump inhibitors, antibiotics, anti-inflammatory drugs, or non-steroidal anti-inflammatory drugs within 4 weeks before the survey; and (iii) no history of gastrectomy. Exclusion criteria comprised: (i) diagnosis of autoimmune disorders (including rheumatoid arthritis and systemic lupus erythematosus); (ii) unwillingness to provide informed consent; and (iii) pregnancy or lactation. All participants provided written informed consent. Gastric biopsies were collected from each patient during upper gastrointestinal endoscopy. Endoscopic examination was performed to assess erosions, atrophy, and intestinal metaplasia. Additionally, we retrieved and analyzed the *iceA* status of *H. pylori* strains from various regions worldwide using data from the NCBI database. A total of 4,449 *H. pylori* whole-genome sequences were obtained, and 1,427 strains were included in the statistical analysis after excluding those lacking source location or patient clinical data.

### *H. pylori* culture conditions

Gastric biopsy specimens obtained from each patient were inoculated onto Skirrow blood agar plates, which contained 5% sheep blood (Yuduo Tech, CHN), 2.5% newborn calf serum (ExCell Bio, CHN), and a combination of antibiotics (Beyotime, CHN), including vancomycin 10 mg/L, polymyxin B 62,000 IU/L, trimethoprim 5 mg/L, amphotericin B 5 mg/L, and cefsulodin 5 mg/L (27). The culture plates were incubated in a microaerobic atmosphere (5% O_2_, 10% CO_2_, and 85% N_2_) for 3–5 days. *H. pylori* colonies, appearing as small, gray, and translucent, were identified and verified through urease, catalase, and Gram staining tests. For gene expression analyses and urea hydrolysis capacity assays, all *H. pylori* strains were cultured in Skirrow broth supplemented with 10% newborn calf serum (ExCell Bio, CHN). Growth kinetics were determined by measuring optical density at 600 nm (OD₆₀₀) until the stationary phase, and cells were harvested specifically during the mid-logarithmic phase (OD₆₀₀ 0.3–0.6).

### Genotype identification of *H. pylori* strains

To identify region-specific genotypes of *H. pylori*, the correlation between *iceA* and *cagA* was examined. For strains isolated from Shenzhen, China, the virulence factors *iceA* and *cagA* were identified using PCR amplification with specific primers (Table 1) (28, 29). Chromosomal DNA was extracted from all colonies of the subculture using the QIAamp DNA extraction kit (QIAGEN, GER), according to the manufacturer’s instructions. The DNA content and purity were assessed using a NanoDrop One spectrophotometer (Thermo Fisher Scientific, USA), and samples were stored at −20℃ before PCR amplification was performed.

**TABLE 1:** PCR primers for amplification of virulence factors

Primer	Primer sequence (5′−3′)	Reference
*iceA1*-F	GTGTTTTTAACCAAAGTATC	30
*iceA1*-R	CTATAGCCAGTCTCTTTGCA	
*iceA2*-F	GTTGGGTATATCACAATTTAT	31
*iceA2*-R	TTRCCCTATTTTCTAGTAGGT	
*cagA* -F	AGGGATAACAGGCAAGCTTTTGA	30
*cagA* -R	CTGCAAAAGATTGTTTGGCAG	
*UreA-*F	TTTCGTTGTCTGCTTGCCTATC	28
*UreA-*R	CGGCTCACACTTCCATTTCTTT	
*UreB-*F	TAACTTCACCCACACGACCCAT	28
*UreB-*R	CCGCTTCCACTAACCCCACTAT	
*16s rRNA*- F	CTCATTGCGAAGGCGACCT	28
*16s rRNA*- R	TCTAATCCTGTTTGCTCCCCA	
*napA*-F	GAATGTGAAAGGCACCGATT	This study
*napA*-R	GGTGATGCCCTAATTGAACG	
*hsp60*-F	GAATTAAGTTGCCCGGTAGC	This study
*hsp60*-R	AGCCGTGATGTTCCTCAAAC	
*omp5*-F	CGATCACCGGTGCGATTG	This study
*omp5*-R	AGGGCTATGACTTGCCAC	
*flaA*-F	GGTTGCGGATAAGGCTAT	This study
*flaA*-R	GATGT*CAGA*TTGAATCGCT	

The amplified DNA products were separated by agarose gel electrophoresis and visualized using a gel imaging system. PCR assays included *H. pylori* strain 26695 as a positive control and a no-template reaction as a negative control. Samples with a mixed *iceA1/iceA2* genotype were excluded from all subsequent analyses to ensure that the results could be unambiguously attributed to a single genotype.

A database comprising 1,427 *H. pylori* genome sequences was constructed from NCBI data for Nucleotide BLAST (BLASTn) analysis. The gene sequences of *H. pylori* strain 26695 (GenBank accession number: AE000511) and strain 11637 (GenBank accession number: LS483488) were selected as the reference for *iceA1* and *iceA2,* respectively, and their target gene sequences were used as queries for comparative analyses. The presence of target genes in these strains was determined by aligning the reference gene sequences against the database genomes, using an *E*-value threshold of 1e-5 and a minimum sequence identity cutoff of 50% to ensure reliable matches. The EPIYA motifs were used to classify *cagA* into two major types: the western type (containing the C motif) and the East-Asian type (containing the D motif) (32, 33). Statistical analyses were conducted on the BLAST results to assess the distribution frequency and conservation of the target genes across the *H. pylori* strains.

### Molecular phylogeny of *iceA* gene based on sequence alignment

To investigate the evolutionary relationships of the *iceA* gene in *H. pylori* strains isolated from patients with different diseases, we conducted sequencing and phylogenetic analysis on strains collected from NCBI. A total of 1,427 strains were included in this study (Table S1). Due to the extremely small sample sizes from Africa and Oceania (only 2 and 5 strains, respectively), strains from these two regions were excluded from further analysis. Consequently, a total of 1,420 strains were retained for subsequent analysis, comprising 836 *iceA1*-positive strains and 584 *iceA2*-positive strains.

The nucleotide and protein sequences of the *iceA1* and *iceA2* genes were aligned using MEGA 12 software to assess sequence similarity, and a maximum likelihood phylogenetic tree was constructed based on genetic distances to infer evolutionary relationships. To ensure the robustness of the phylogenetic tree, bootstrap analysis with 1,000 replicates was performed, evaluating the stability and reliability of the tree topology. Divergence among the samples was subsequently exported to the Interactive Tree Of Life platform for advanced visualization and annotation.

### Analysis of gene expression

To investigate the impact of the *iceA* gene on gene expression in *H. pylori*, reverse transcription quantitative PCR was performed using the SYBR Green dye method. The study included 13 *iceA1*-positive and 12 *iceA2*-positive *H. pylori* strains isolated from Shenzhen, China. RNA was extracted from *H. pylori* using acid guanidinium thiocyanate–phenol–chloroform (TRIZOL) method (28, 34), and its quality was assessed by gel electrophoresis and spectrophotometry. The LightCycler 480 (Roche, SUI) was used for quantitative PCR, with primers designed to amplify specific amplicons (Table 1). Melting curve analysis was performed to confirm the specificity of the products. *H. pylori* 16S rRNA served as the internal reference gene, and the 2^−ΔΔCt^ method was used to determine relative gene expression. No-reverse transcription controls were included in all runs to rule out genomic DNA contamination. Each sample was run in triplicate, and the experiment was repeated three times (35).

### Urea hydrolysis test of *H. pylori*

Urea hydrolysis assay was performed using a urease reagent kit containing urea and phenol red (pH indicator). Urease-catalyzed urea hydrolysis increases pH, inducing a color shift from yellow to purple-red, allowing quantitative measurement of enzyme activity (36).

*H. pylori iceA1*-positive and *iceA2*-positive strains were cultured to the logarithmic growth phase, harvested, and resuspended in reaction buffer. After adding the urease reagent, absorbance at 560 nm was continuously monitored using a microplate reader. Time-absorbance curves were plotted to compare urea hydrolysis capacity between the two groups.

### Statistical analysis

All statistical analyses were conducted using GraphPad Prism 9.0.0 software. Data were analyzed using Student’s *t* tests or Fisher’s exact tests. Two-sided *P*-values < 0.05 were considered statistically significant. Statistical significance in the figures is denoted as follows: ns (not significant), *P < 0.05, **P < 0.01, ***P < 0.001, and ****P < 0.0001.

## RESULTS

### Regional diversity in the distribution of *iceA* gene genotypes

**Fig 1:**
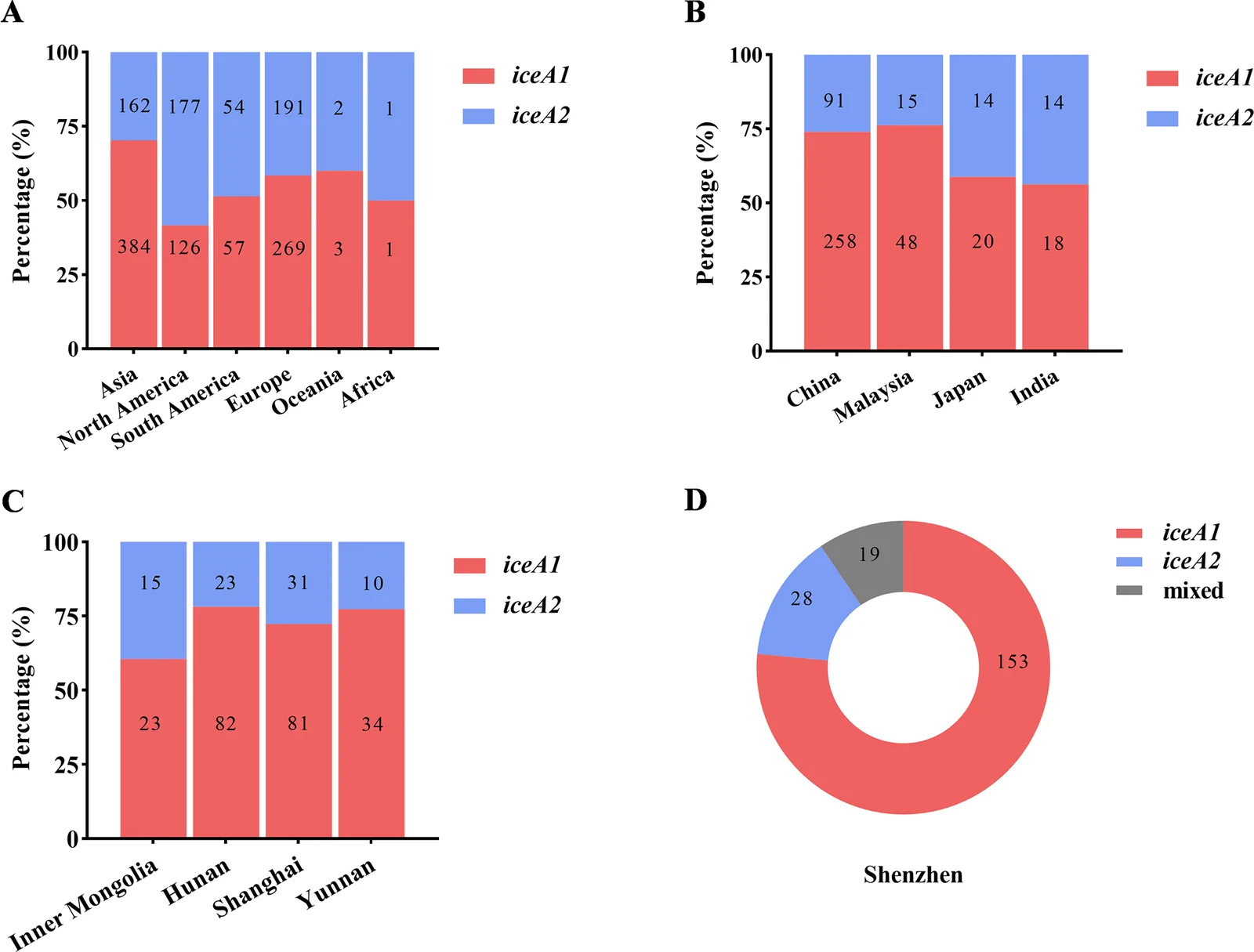
Distribution of *iceA* gene subtypes in *H. pylori* strains across different geographical regions. (**A**) Distribution proportions of *iceA1* and *iceA2* subtypes among *H. pylori* strains isolated from different continents, including Asia, North America, South America, Europe, Oceania, and Africa. (**B**) Detailed distributions within selected countries in Asia. Panels **C and D** further examine the distribution within specific regions in China. The numbers inside the bars represent the sample size (*N*) for each genotype.

A total of 1,427 *H. pylori* strains from the NCBI database were analyzed (Fig. 1). The strains were predominantly from Asia (*n* = 546) and Europe (*n* = 460), followed by North America (*n* = 303), South America (*n* = 111), with a minimal number of samples from Oceania (*n* = 5) and Africa (*n* = 2). The distribution of *iceA* genotypes showed significant regional variation. In Asia and Europe, *iceA1* was the predominant genotype.

The study also included 200 samples from Shenzhen, China. Of these, 181 were infected with single *iceA* genotype strains, while 19 samples were co-infected with both *iceA1* and *iceA2* strains. Among patients infected with a single *iceA* genotype in Shenzhen, *iceA1* was the predominant genotype, accounting for 84.53% of cases (*n* = 153), compared to 15.47% (*n* = 28) for the *iceA2* genotype (Fig. 1D). The prevalence of *iceA1* in Shenzhen was similar to that in the broader Asian region. In contrast, *iceA2* was more prevalent than *iceA1* in North America. Interestingly, in South America, the two genotypes were approximately equally distributed, each accounting for about half of the cases. These findings highlight the geographical heterogeneity in *iceA* genotype distribution, with distinct patterns observed between Asian, European regions, and the Americas.

### Distribution of *iceA* genotype in relation to *cagA*

**Fig 2:**
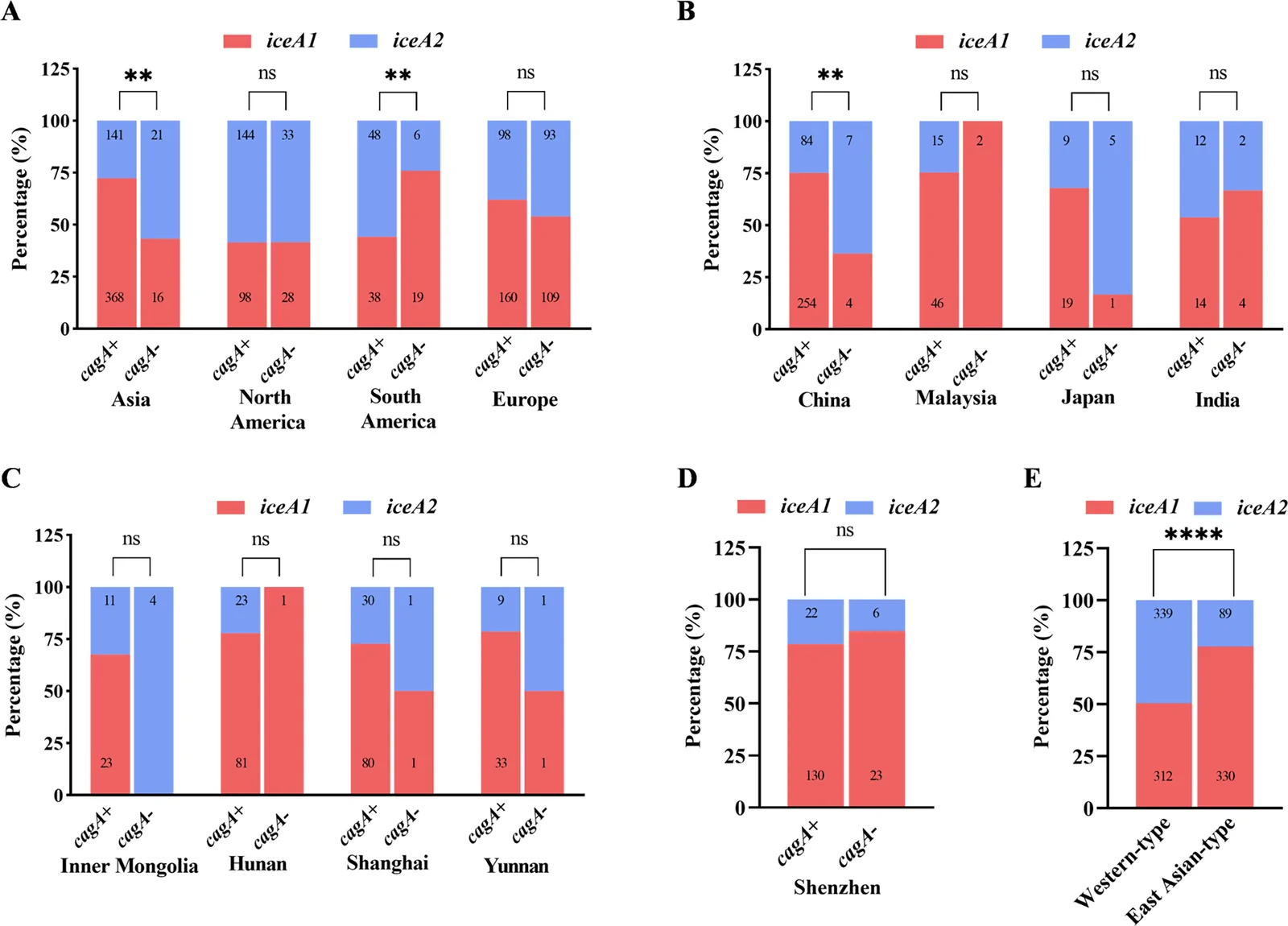
Association analysis of *iceA* genotypes with *cagA* gene in *H. pylori* strains across different geographical regions. (**A**) Correlation between *iceA* genotypes and *cagA* status in *H. pylori* strains isolated from Asia, North America, South America, and Europe. (**B**) Analysis of *iceA* genotypes and *cagA* status in *H. pylori* strains from various countries in Asia. (**C and D**) Analysis of *iceA* genotypes and *cagA* status in *H. pylori* strains from different regions in China. (**E**) Correlation between *iceA* genotypes and *cagA* types. The numbers inside the bars represent the sample size (*N*) for each genotype. Statistical analysis was performed using two-sided *χ*^2^ tests with Fisher’s exact test. Significant associations are indicated by asterisks: ns (not significant), *P* ≥ 0.05, **P < 0.01, and ****P < 0.0001.

The association between *iceA* genotypes and *cagA* status exhibited distinct regional patterns (Fig. 2). In Asia, *iceA1* was positively correlated with *cagA*, with *iceA1* strains demonstrating a higher *cagA* positivity rate compared to *iceA2* strains. Conversely, in South America, *iceA2* strains exhibited a higher *cagA* positivity rate than *iceA1* strains. Data from North America and Europe revealed no significant correlation between *iceA* genotypes and *cagA* status. In the Shenzhen region, analysis of strains with single *iceA* genotype infections showed no significant correlation between *iceA* genotypes and *cagA* status. However, the overall trend in Shenzhen appeared similar to that observed in the broader Asian region. These findings highlight the geographical variability in the association between *iceA* genotypes and *cagA* status, suggesting potential regional differences in *H. pylori* virulence factor interactions.

Although the correlation between *iceA1* and *cagA* positivity was inconsistent across regions, we observed a pronounced association between *iceA* genotypes and *cagA* types. Specifically, *iceA1* was present in approximately half of the western-type *cagA* strains but accounted for nearly 80% of the East Asian-type *cagA* strains—a significantly higher proportion (Fig. 2E), indicating a notable connection between the *iceA1* genotype and the East Asian-type *cagA*.

### Evolutionary relationships of *iceA1* gene variants in global *H. pylori* isolates

**Fig 3:**
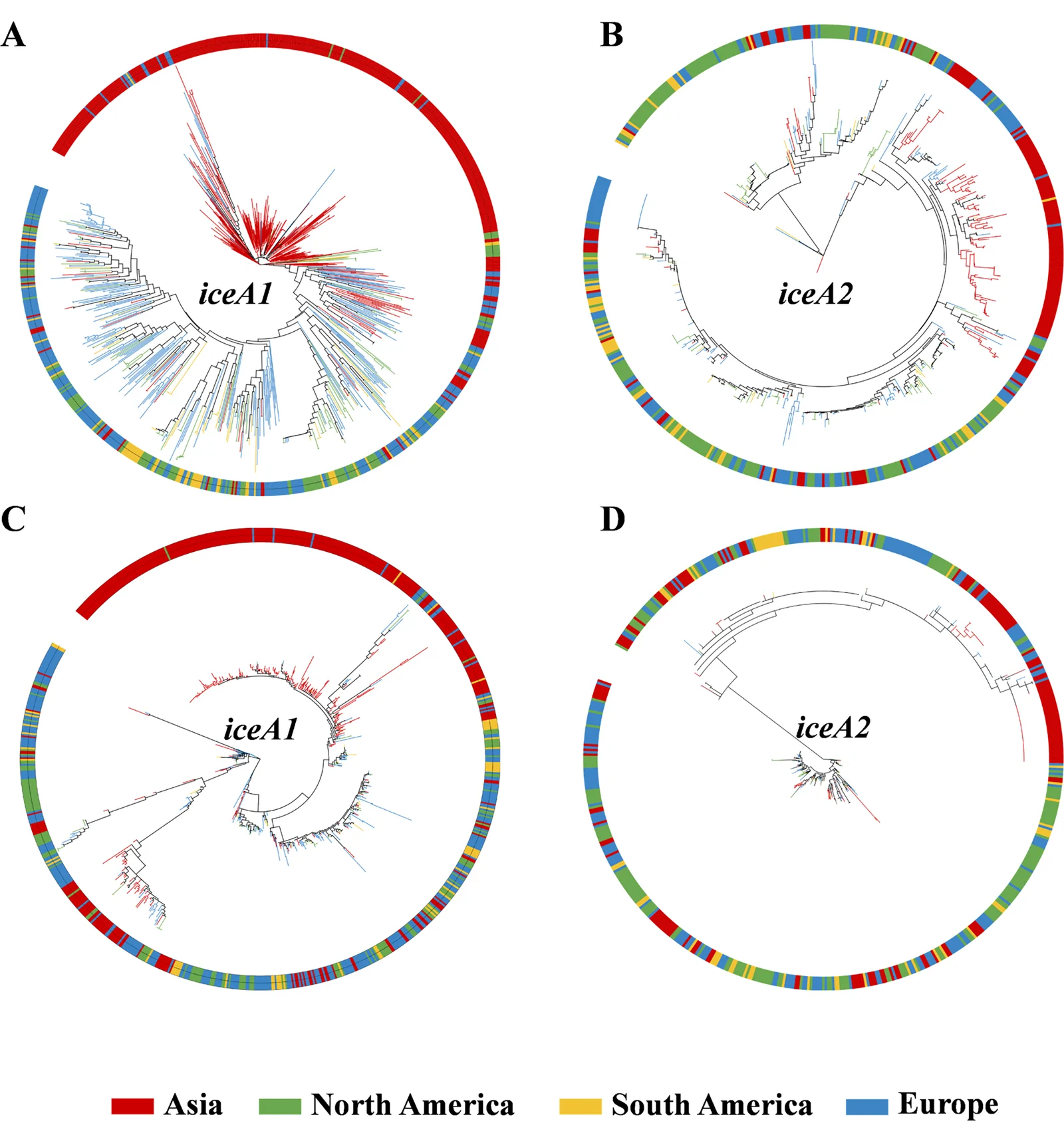
Phylogenetic tree of *iceA1* (*n* = 836) and *iceA2* (*n* = 584) gene sequences from *H. pylori* strains. Strains are color coded by geographic origin: red for Asia, green for North America, yellow for South America, and blue for Europe. Phylogenetic trees of the *iceA1* and *iceA2* genes based on (**A and B**) nucleic acid sequences and (**C and D**) protein sequences.

The phylogenetic tree based on the *iceA1* and *iceA2* gene and protein demonstrated distinct geographic clustering of *H. pylori* strains (Fig. 3). Both genes consistently demonstrated that Asian strains formed a highly supported monophyletic clade, exhibiting remarkable genetic distinctiveness with significantly lower nucleotide diversity compared to other regions, suggesting potential long-term geographic isolation or host-specific adaptive evolution in this area. In contrast, North American, South American, and European strains showed extensive admixture across the phylogenetic tree without clear geographic clustering, suggesting frequent strain exchange and genetic mixing among these regions. Notably, both *iceA1* and *iceA2* from Asian strains formed distinct phylogenetic clusters compared with strains from other continents, indicating significant genetic differentiation from strains of other geographic regions. The phylogenetic trees constructed for the *iceA1* and *iceA2* genes at both nucleotide and protein sequence levels shared several consistent features. For example, strains from the same geographical origin exhibited comparable clustering patterns in both nucleotide- and protein-based trees. However, differences existed in details such as branch density, branch arrangement, and the degree of relatedness between lineages. Such consistency and differences may be caused by various factors, such as selection pressure, protein structure, and functional constraints, after nucleic acid sequence variations are translated into proteins.

### *IceA* status is associated with *H. pylori ureA* gene expression levels

**Fig 4:**
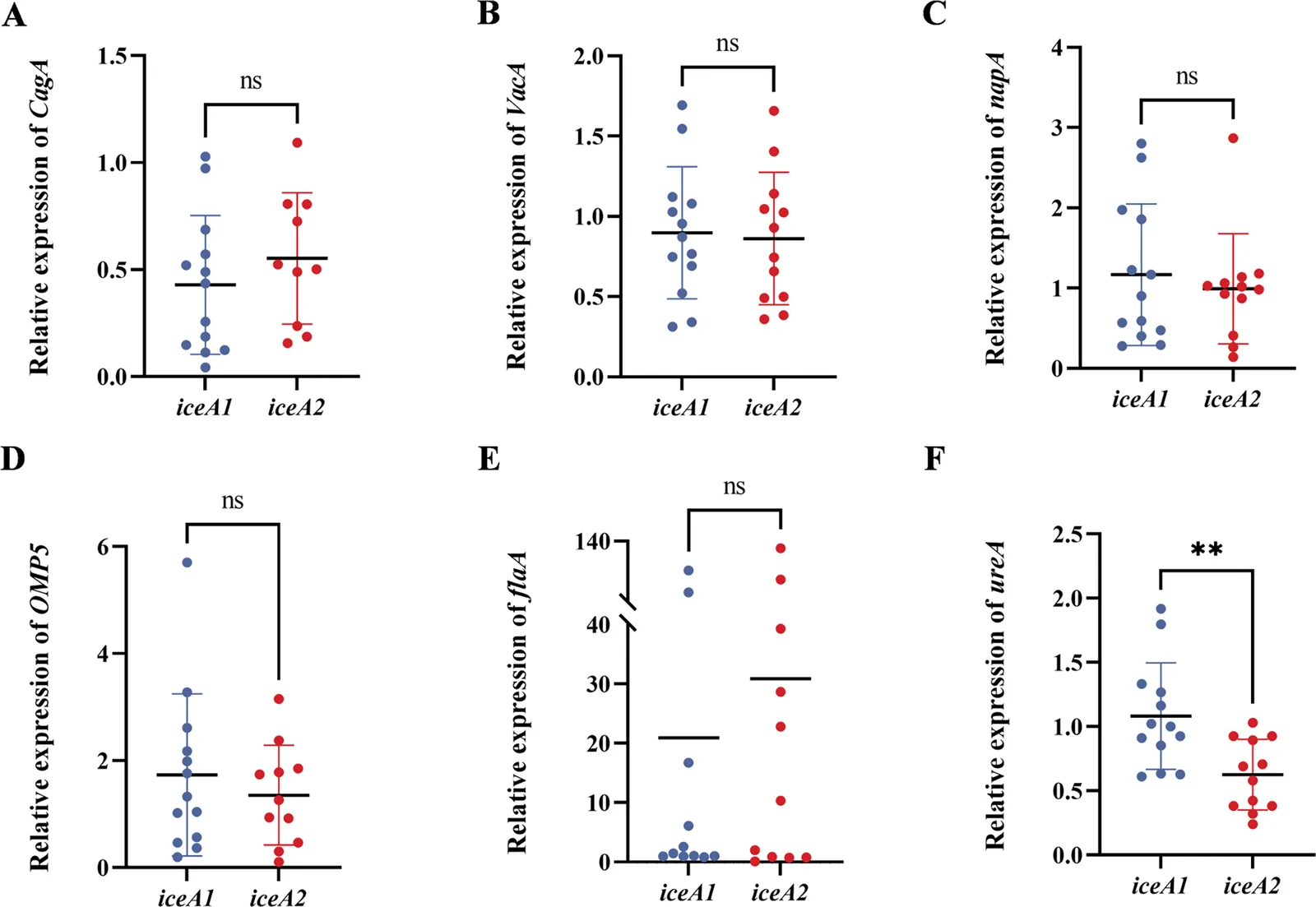
Comparative gene expression analysis in *iceA1*-positive and *iceA2*-positive *H. pylori* strains. Panels A–F depict the expression levels of the following genes: (**A**) *cagA*, (**B**) *vacA,* (**C**) *napA*, (**D**) *OMP5*, (**E**) *flaA*, and (**F**) *ureA*. Each data point represents the mean relative gene expression level of the corresponding gene in strains of the indicated genotype. Error bars represent the standard deviation (SD) from three independent biological replicates performed on strains of the same genotype. Statistical analysis was performed using two-sided *t*-tests (or Mann-Whitney *U* tests if data were not normally distributed). Data represent the median from triplicate cultures. ns, not significant and **P < 0.01.

To investigate the potential regulatory effect of *iceA* on gene expression in *H. pylori*, expression analysis was performed using clinical isolates from the Shenzhen region (Fig. 4). The study focused on key virulence factors encoding various pathogenic proteins: cytotoxin-associated gene A (*cagA*), vacuolating cytotoxin A (*vacA*), urease A (*ureA*), neutrophil activating protein A gene (*napA*), outer membrane protein 5 gene (*OMP5*), and flagellin A (*flaA*). These factors are associated with cytotoxicity, acid resistance, inflammation induction, adhesion, and colonization.

**Fig 5:**
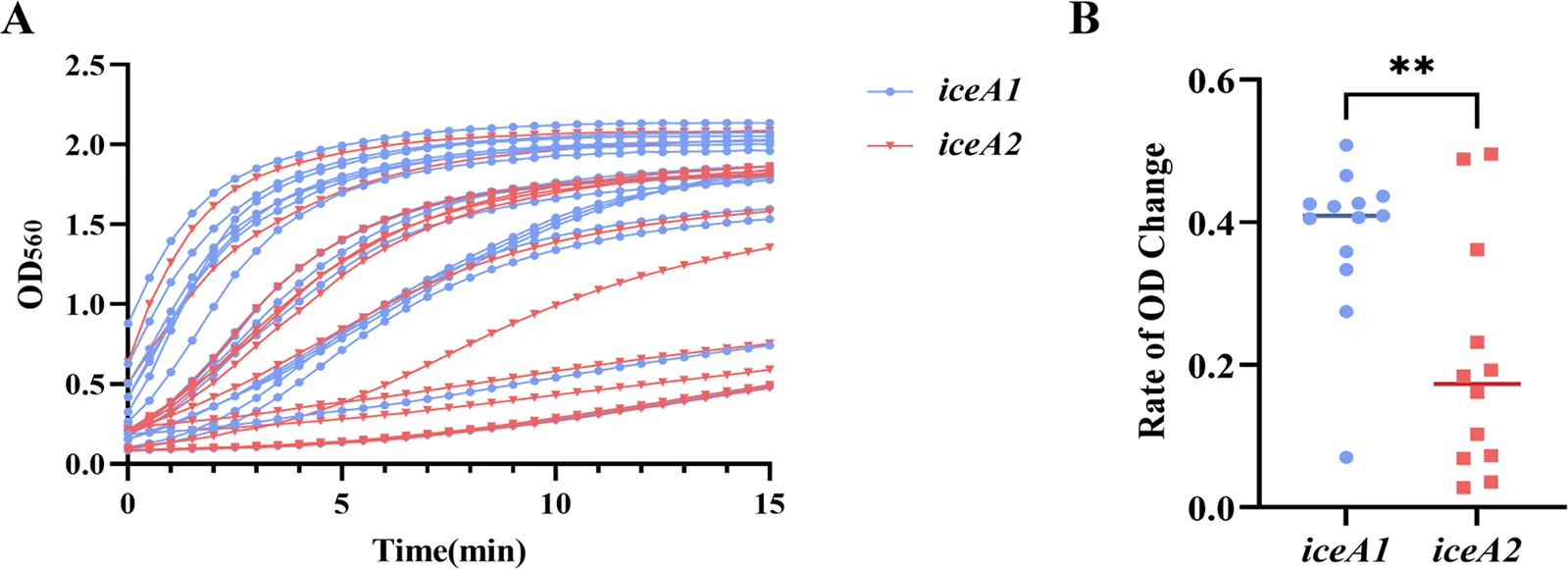
Urea hydrolysis capacity between *iceA1* and *iceA2* genotypes of clinical *H. pylori* strains: (**A**) time-course measurement of urea hydrolysis, showing continuous absorbance values over time. (**B**) Statistical comparison of urea hydrolysis kinetics between *iceA1*- and *iceA2*-positive strains. Each data point represents the mean absorbance value at the indicated time point. Error bars represent the standard deviation (SD) of three independent replicates. The rate of absorbance change during the initial 1-minute reaction phase was calculated and analyzed by *t*-tests. **P < 0.01.

The results revealed a significant association between *iceA* genotypes and the expression of certain virulence factors. Specifically, *iceA1*-positive strains exhibited significantly higher expression levels and urea hydrolysis capacity of *ureA* compared to *iceA2*-positive strains (Fig. 5). However, the expression levels of *cagA*, *vacA*, *napA*, *OMP5*, and *flaA* did not show significant correlations with *iceA* genotypes (*P* > 0.05). The absence of significant correlations between *iceA* genotypes and the expression of these virulence factors suggests that other strain-specific genetic factors may primarily modulate these pathogenic determinants. These results indicate that while *iceA* may be associated with *ureA* expression, its impact on other key virulence factors appears limited in the studied population.

### Analysis of the correlation between *iceA* and peptic ulcer

**Fig 6:**
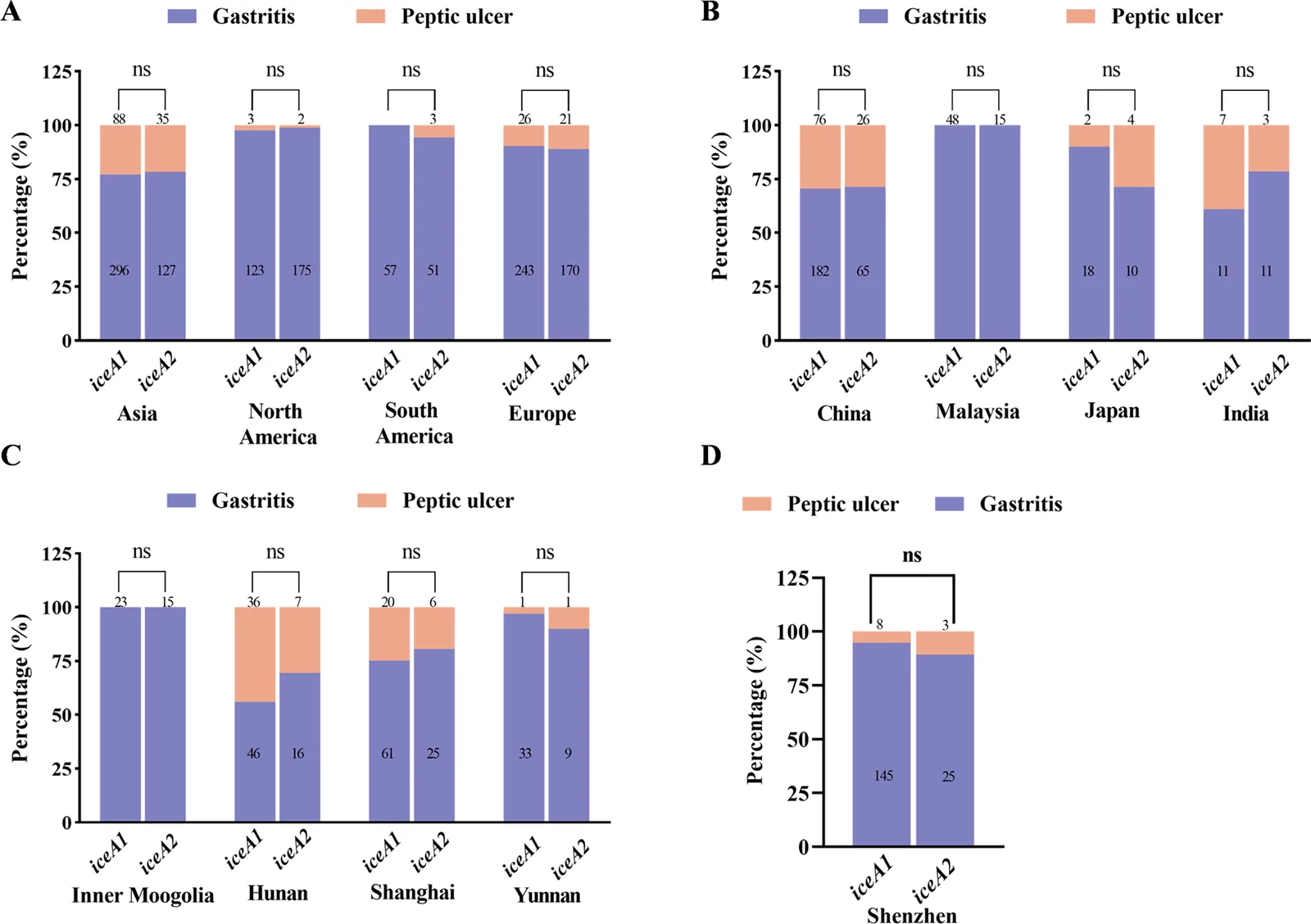
Association between *iceA* genotypes and gastrointestinal diseases across different geographical regions. (**A**) Prevalence of peptic ulcer disease in patients infected with *iceA1*-positive or *iceA2*-positive *H. pylori* strains in different continents, including Asia, North America, South America, and Europe. (**B**) Detailed analyses within selected countries in Asia. (**C and D**) Further examines the associations within specific regions in China. The numbers inside the bars represent the sample size (*N*) for each genotype. Statistical analysis was performed using two-sided *χ*^2^ tests with Fisher’s exact test. ns, not significant.

Previous studies have indicated that infection with *iceA1*-positive *H. pylori* strains may correlate with an increased risk of chronic gastritis and peptic ulcer disease (14, 21). However, our global analysis demonstrated no significant correlation between *iceA* genotypes and peptic ulcer disease across diverse regions (Fig. 6). These findings suggest that the relationship between *iceA* genotypes and gastric diseases may be more complex than previously thought and could vary among different populations or geographical regions.

### *IceA1* gene in association with chronic gastritis and mucosal erosion

Since the majority of *H. pylori* infections progress only to gastritis without further complications, we specifically investigated whether the presence of the *iceA1* gene was associated with particular gastric histopathological features among patients diagnosed with chronic gastritis, aiming to identify potential molecular markers for disease progression. We performed a detailed analysis of endoscopic pathological features for the *H. pylori* strains isolated from the Shenzhen region. Among these, 19 cases were co-infected with both *iceA* and *iceA2* genotypes, while 181 cases harbored a single *iceA* genotype. We further investigated the association between *iceA* genotypes and endoscopic pathological features in these 181 gastritis patients, assessing three key indicators: presence or absence of (i) erosions, (ii) atrophy, and (iii) intestinal metaplasia (Table 2). Statistical analysis was performed using two-sided *χ*^2^ tests or Fisher’s exact test, as appropriate. The results indicated that there was no significant correlation between *iceA* genotypes and the severity of gastric atrophy or intestinal metaplasia. However, it was interesting to note that patients infected with *iceA1* genotype strains were more likely to present with erosive chronic gastritis, whereas those infected with *iceA1*-negative strains were more likely to present with non-erosive chronic gastritis (*P* < 0.05; Table 2).

**TABLE 2:** Association between *iceA* genotypes and gastric erosion in *H. pylori* strains from Shenzhen, China

Clinical status		*iceA1, n* (%)	*iceA2, n* (%)	*P* value
Erosion status	Non-erosive	102 (80.95)	24 (19.05)	0.03
	Erosive	51 (92.73)	4 (7.27)	
Atrophy status	Non-atrophic	131 (85.06)	23 (14.94)	0.58
	Atrophic	22 (81.48)	5 (18.52)	
Metaplasia status	Without intestinal metaplasia	124 (86.11)	20 (13.89)	0.31
	Intestinal metaplasia	29 (78.38)	8 (21.62)	

## DISCUSSION

In this study, we investigated the regional distribution of *iceA* genotypes in *H. pylori* strains and their associations with virulence markers, gene expression, and gastric disease. Analysis of 1,427 strains from the NCBI database showed that *iceA1* is predominant in Asia and Europe, while *iceA2* is more prevalent in North America. The association between *iceA* genotypes and *cagA* status also varied regionally, with *iceA1* positively correlated with *cagA* in Asia and *iceA2* showing higher *cagA* positivity in South America. In the Shenzhen region, *iceA1* was associated with higher *ureA* expression, potentially enhancing bacterial colonization. Importantly, our analysis of clinical isolates from Shenzhen demonstrated that patients infected with *iceA1* genotype strains were more likely to present with erosive chronic gastritis. Our findings highlight the geographical heterogeneity of the *iceA1* genotype and its potential role in the early stages of gastric disease.

The analysis of the geographical distribution of the *iceA* genotype among *H. pylori* strains reveals significant global disparities that likely reflect pathogen adaptation to diverse human populations (11, 37–39). The phylogenetic tree analysis demonstrates distinct evolutionary patterns: Asian *iceA1* strains form a monophyletic cluster with significantly lower nucleotide diversity, suggesting long-term geographical isolation and possible co-evolution with local hosts. This phylogenetic evidence supports the observed high prevalence of *iceA1* in Asia (40–43). Conversely, the predominance of the *iceA2* genotype in North America may be attributed to region-specific environmental pressures or population migration history. In contrast, the extensive phylogenetic intermixing of North American, European, and South American strains correlates with their balanced genotype distribution. Particularly in South America, the coexistence of *iceA1* and *iceA2* genotypes likely results from historical population admixture, as our phylogenetic data show. These findings reveal the global distribution characteristics of the *iceA* gene and provide insights into the geographical adaptability processes of *H. pylori*.

Previous studies have elucidated that the *iceA* gene functions as an independent virulence factor in the etiology of gastric diseases (21, 37). However, our research indicates that the correlation between *iceA* and *cagA* varies across different geographical regions. This suggests that *iceA* may not function as a completely independent pathogenic factor. Instead, its role may be modulated by geographical environment, host factors, or interactions with other genes. The correlation between *iceA1* and *cagA* positivity varied geographically. This variation may reflect regional differences in bacterial population structure or host genetics. The striking discrepancy in *iceA1* prevalence between western-type and East Asian-type *cagA* strains (approximately 50% and 80%, respectively) suggests that the association between *iceA* and *cagA* may be driven not merely by the presence of the *cagA* gene, but rather by deep-rooted co-evolution within specific bacterial lineages. This indicates that the *iceA1* allele might be genetically linked or functionally co-adapted with the East Asian-type *cagA* pathogenicity island, possibly contributing to their synergistic role in virulence or niche adaptation in certain host populations. This phenomenon may be attributed to the distinct evolutionary pressures faced by *H. pylori* in various regions, leading to the natural selection of region-specific dominant strains. These strains likely possess unique genomic structures and regulatory mechanisms, thereby altering the relationships between virulence genes.

*IceA1*, which encodes a nuclease, may play a role in regulating gene expression (44). Previous studies have shown that *iceA* gene subtypes are correlated with the transcription levels of the downstream gene-*hpyIM* methylase gene (16, 45). However, research on *iceA*’s regulatory effects on other genes has been limited. Our study demonstrates that *iceA1*-positive strains exhibit higher expression levels of *ureA* and higher urea hydrolysis capacity compared to *iceA2* strains. The *urea*se encoded by the *ureA* is essential for *H. pylori*’s survival in the acidic gastric environment. The presence of *iceA1* may enhance the bacteria’s adaptability to acidic conditions, thereby increasing their pathogenicity. This finding suggests a functional synergy between *iceA1* and *ureA* in *H. pylori*’s pathogenesis and highlights the complex gene regulatory network within this bacterium.

From an evolutionary perspective, the association between *iceA1* and *ureA* expression may confer a survival advantage to *iceA1*-positive strains in specific gastric microenvironments. This discovery has significant clinical implications. The relationship between *iceA1* and *ureA* expression could serve as a novel indicator of *H. pylori* infection severity. It not only deepens our understanding of *H. pylori*’s pathogenic mechanisms but also provides new insights into the bacterium’s geographical adaptability and evolutionary processes. This provides valuable insights into the specific regulatory functions of *iceA* in *H. pylori* strains, particularly its association with urease production. It is crucial to note that these findings are derived from *in vitro* experiments, which cannot fully replicate the complex host-pathogen interactions within a living organism. To truly validate the proposed survival advantage and its impact on pathogenicity, future research employing animal models or co-culture with human gastric epithelial cells is essential. Besides, the lack of correlation with other virulence factors suggests that *iceA*’s role in *H. pylori* pathogenesis may be more complex than previously thought. Further investigation into its mechanisms of action and potential indirect effects on bacterial virulence is warranted.

Although some studies suggest a potential association between *iceA* genotype and gastric disease (14, 15, 21), our comparison between gastritis and control groups revealed no correlation between the *iceA1* genotype and an increased risk of peptic ulcer disease or gastric cancer, consistent with prior research (15, 24, 25, 46, 47). By analysis of endoscopic findings, our results revealed a significant association between the presence of *iceA1* and an increased risk of erosive gastritis in the Chinese population. Given that gastric erosion is considered a precursor to gastric ulcer and that erosive gastritis may progress to gastric ulcer, the observed association suggests a potential early pathogenic role for *iceA1* (48). The significant correlation between *iceA1* and erosive gastritis in China may reflect the influence of specific environmental factors or host genetic backgrounds unique to this population. Alternatively, *iceA1* may play a more critical role in the initial stages of gastric disease, such as erosive gastritis, while its impact on later stages, like peptic ulcer or gastric cancer, may be obscured by other factors. These findings highlight a possible link between the *iceA1* genotype and the development of erosive gastritis, providing new insights into the pathogenesis of *H. pylori*-related gastric diseases.

Although this study provides a comprehensive analysis of *H. pylori* strains from global regions, the data sourced from the NCBI database have limitations. Specifically, the database used lacks information on whether the strains were involved in mixed infections, which could potentially mask some results. Additionally, the database does not provide detailed information regarding gastric pathology, such as grading information and metaplasia, nor does it include data on erosive conditions. Furthermore, our correlation analysis between *iceA* and virulence genes was restricted to *cagA*; other functionally important genes like *vacA*, *babA*, and *dupA* remain unexamined, warranting future investigation to elucidate their potential interactions. It should be noted that in the present study, *CagA* was only classified into Western and East Asian types, and further refined subtyping of Western *CagA* variants (e.g., ABC, ABCC, and ABCCC) was not performed. Such detailed subtyping has important biological and clinical implications, as it may help to better elucidate the pathogenic heterogeneity and diverse clinical outcomes linked to different *CagA* structural variants. Therefore, future studies will be conducted to explore detailed *CagA* subtyping and its associations with clinical phenotypes and disease progression. Future research should aim to address these gaps by incorporating data on mixed infections and providing more comprehensive details on gastric pathology. This would enhance the interpretation of *H. pylori*’s impact on health. In the analysis of the relationship between *iceA* and *ureA* gene expression, our study has a deficiency in the adaptability between the strength of evidence and the statement of conclusions: the existing data can only confirm the correlation between the two; the expression level of *ureA* is relatively higher in strains carrying the *iceA1* genotype, but cannot support the conclusion that “*iceA* regulates *ureA* expression.”

To address this limitation, subsequent studies are needed to clarify the functional association between the two through targeted experiments. Specifically, an *iceA1* gene knockout mutant (∆*iceA1*) will be constructed, or alternatively, *iceA1*/*iceA2* gene swapping will be performed in strains with similar genetic backgrounds but different *iceA* genotypes. This will rule out the interference of strain background and provide direct evidence for the regulatory relationship between *iceA* and *ureA*.

### Conclusion

This study reveals that *iceA* genotypes display distinct regional variations and exhibit differential correlations with other virulence genes based on geographic context. Besides, *iceA1* was found to be associated with the expression of *ureA*, which may enhance bacterial colonization and contribute to gastric mucosal damage during the early stages of infection, thereby increasing the risk of gastric erosion. These insights advance our understanding of the pathogenic mechanisms of *H. pylori* and underscore the potential role of *iceA1* in the progression of gastric diseases.

## SUPPLEMENTAL MATERIAL

Table S1 spectrum.02024-25-s0001.xlsx Data on strains retrieved from NCBI.

ASM does not own the copyrights to Supplemental Material that may be linked to, or accessed through, an article. The authors have granted ASM a non-exclusive, world-wide license to publish the Supplemental Material files. Please contact the corresponding author directly for reuse.
